# Dysregulation of RIG-I activation by picornavirus 3A protein

**DOI:** 10.1128/jvi.02069-25

**Published:** 2026-05-20

**Authors:** Xiangle Zhang, Zhenxiang Zhao, Kangli Li, Wenzhe Chen, Guanshun Wang, Fan Yang, Guoliang Zhu, Jijun He, Xi Lan, Haixue Zheng, Pengfei Li, Zixiang Zhu

**Affiliations:** 1 State Key Laboratory of Veterinary Etiological Biology, College of Veterinary Medicine, Lanzhou University, Lanzhou Veterinary Research Institute, Chinese Academy of Agricultural Sciences 12426, Lanzhou, China; 2 Department of Medicine, Washington University School of Medicine 12275 , St. Louis, Missouri, USA; Fred Hutchinson Cancer Center Vaccine and Infectious Disease Division, Seattle, Washington, USA

**Keywords:** interferon signaling, RIG-I, 3A, picornavirus

## Abstract

Retinoic acid-inducible gene I (RIG-I)-like receptors, including RIG-I and MDA5, are key cytoplasmic pattern-recognition receptors that detect viral RNA in the cytoplasm and trigger the production of interferon (IFN). Among them, MDA5, rather than RIG-I, is considered the primary sensor during picornavirus infection due to the unique features of picornaviral RNA. However, previous studies have indicated that RIG-I also possesses antiviral activity against several picornaviruses, suggesting its potential importance during viral replication. Here, we found that 3A proteins from various picornaviruses, including Senecavirus A (SVA), Enterovirus 71 (EV71), Encephalomyocarditis virus (EMCV), foot-and-mouth disease virus (FMDV), and Coxsackievirus A16 (CA16), directly target RIG-I to suppress RIG-I-like receptor-mediated IFN-β production. Mechanistically, these picornaviral 3A proteins partially share the following similar strategies to dysregulate RIG-I activation: (i) all interact with RIG-I, (ii) EMCV and FMDV 3A reduce RIG-I expression, and (iii) SVA, EV71, EMCV, and FMDV 3A impair the interaction between RIG-I and MAVS by diminishing K63-linked ubiquitination of RIG-I. These findings broaden our understanding of how picornaviruses employ nonstructural proteins to evade innate immune responses during early infection.

**IMPORTANCE:**

Picornaviruses cause a broad spectrum of human and animal diseases; however, the mechanisms by which they counteract host antiviral defenses remain incompletely understood. Owing to the distinct structural features of picornaviral RNA, MDA5 is widely regarded as the primary sensor mediating antiviral responses during picornavirus infection. However, accumulating evidence suggests that RIG-I also contributes to antiviral defense. Picornaviruses have evolved various means to suppress RIG-I and MDA5 activity, thereby facilitating evasion of the innate immune response and underscoring the importance of RIG-I during picornavirus infection. This study identifies RIG-I as a conserved target of the nonstructural protein 3A from multiple picornaviruses, including Senecavirus A, Enterovirus 71, Encephalomyocarditis virus, foot-and-mouth disease virus, and Coxsackievirus A16, and uncovers both shared and virus-specific strategies that dysregulate RIG-I-mediated interferon production. Collectively, these findings expand our understanding of the antagonistic mechanisms of how picornaviruses manipulate the host innate immune system.

## INTRODUCTION

Retinoic acid-inducible gene I (RIG-I)-like receptors (RLRs) are a family of key intracellular RNA sensors that detect viral RNA and trigger the production of type I and type III interferons, establishing an antiviral state that restricts viral infection. The RIG-I-like receptor (RLR) family encompasses three members: RIG-I, melanoma differentiation-associated factor 5 (MDA5), and laboratory of genetics and physiology 2 (LGP2). RIG-I and MDA5 serve as primary cytoplasmic sensors of viral RNA, whereas LGP2 acts as a regulatory protein that negatively regulates the activity of RIG-I and MDA5 (1). It is well established that RIG-I and MDA5 recognize different RNA ligands; RIG-I primarily binds to short double-strand RNA (dsRNA) as well as single-stranded RNA (ssRNA) containing 5′-triphosphate moieties, while MDA5 preferentially senses long ssRNA. Therefore, RIG-I and MDA5 function in different types of viral infections, with RIG-I preferentially recognizing viruses from the families *Orthomyxoviridae*, *Bunyaviridae*, and *Arteriviridae*, whereas MDA5 primarily detects the members of *Reoviridae, Coronaviridae,* and *Picornaviridae* (1). Notably, some viruses can be sensed by both RIG-I and MDA5, such as West Nile virus (WNV) from the *Flaviviridae* family and measles virus from the *Paramyoviridae* family (1, 2).

Picornaviruses are a highly diverse group of RNA viruses that cause a wide range of diseases in humans and animals, such as *Enterovirus*, *Coxsackievirus*, *Cardiovirus*, *Aphthovirus*, and *Senecavirus* (3). Picornaviruses possess a single-stranded, approximately 7–9 kb, positive RNA genome containing an open reading frame flanked by 5′ and 3′ untranslated regions. The 5′ end of the viral genome lacks the typical cap structure and instead contains a covalently attached virus-encoded protein known as VPg, which enables picornaviruses to initiate protein translation through a cap-independent mechanism (4). Picornavirus replication also generates a single-stranded, negative-sense RNA intermediate that serves as a template for the synthesis of the progeny viral genome. These features render picornaviruses more readily detected by MDA5 rather than RIG-I, positioning MDA5 as the primary sensor and frequent target of viral antagonism. However, recent studies indicate that RIG-I also plays a critical role in activating the interferon response during picornavirus infection and can likewise be targeted by picornaviruses (5), which have evolved various strategies to evade RIG-I-mediated antiviral defense (6). The open reading frame of picornaviruses encodes a viral polyprotein that is proteolytically cleaved to four structural proteins (VP1, VP2, VP3, and VP4) and eight non-structural proteins (L, 2A, 2B, 2C, 3A, 3B, 3C, and 3D) (7). The 3A protein is crucial for picornavirus replication, playing essential roles in the formation of the viral replication complex and virus-induced replication organelles, as well as determining the host range (8). In addition, some picornaviral 3A proteins disrupt protein trafficking, impairing MHC-mediated antigen presentation on the cell surface and inhibiting the secretion of immune regulatory proteins (9–11). Furthermore, 3A protein antagonizes the innate immune response to facilitate virus replication (12–15).

Our previous studies reported that FMDV 3A employs multiple mechanisms to inhibit IFN-β production by interfering with RIG-I activity, including direct interaction or indirect modulation of other pathways (12, 14, 15). These findings prompted us to further investigate the effects of other picornaviral 3A proteins on the innate immune response. In this study, we found that 3A proteins from a series of picornaviruses, including SVA, EV71, EMCV, and CA16, target RIG-I and negatively regulate its activity through similar, yet distinct mechanisms to suppress IFN-β production. These data highlight the important role of picornaviral 3A protein in blocking interferon response, a strategy that is critical for evading host innate immune response and establishing infection during the early stage of the viral life cycle.

## RESULTS

### SVA 3A protein suppresses IFN-β production

SVA is the sole member of the genus *Senecavirus* within the family *Picornaviridae* and is known to cause idiopathic vesicular disease in swine (16, 17). SVA infection has been reported to suppress innate immune response (18). We previously found that SVA 3A protein antagonizes the RLR signaling pathway and inhibits activation of the IFN-β promoter (19). In this study, we further sought to investigate the underlying mechanisms by which SVA 3A protein suppresses type I interferon production.

**Fig 1:**
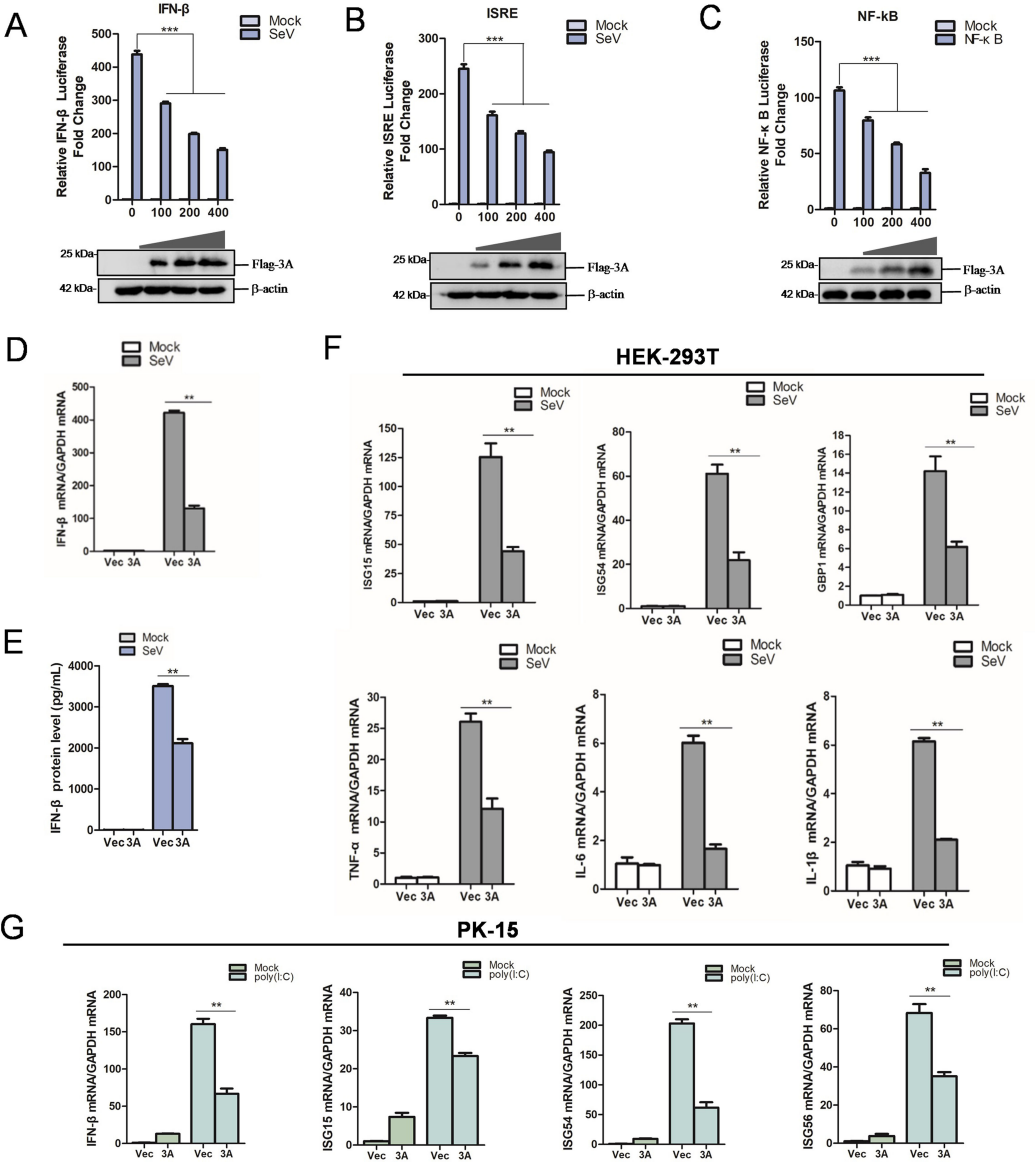
Inhibition of IFN-β and ISGs production by the SVA 3A protein. (**A–C**) HEK-293T cells were transfected with the indicated reporter plasmids (50 ng each): IFN-β (**A**), ISRE (**B**), and NF-κB (**C**), the PRL-TK control reporter (5 ng), and increased doses (0, 100, 200, or 400 ng) of plasmids expressing SVA 3A for 24 h, followed by treating with Sendai virus (SeV) for 12 h. Cell lysates were collected for measuring luciferase activity using a dual luciferase assay. (**D and E**) HEK-293T cells were transfected with an empty vector or SVA 3A expressing plasmids for 24 h, prior to treatment with SeV for 12 h. Cell lysates were processed to detect IFN-β mRNA levels by qPCR (**D**), and the supernatants were collected to measure IFN-β protein levels (**E**). (**F**) HEK-293T cells were transfected with an empty vector or a plasmid expressing SVA 3A. After 24 h, the transfected cells were treated with SeV for another 12 h. Cell lysate was harvested to evaluate the mRNA levels of ISGs, including ISG15, ISG54, GBP1, TNF-α, IL-6, and IL-1β, using qPCR. (**G**) PK-15 cells were transfected with an empty vector or a plasmid expressing SVA 3A for 24 h, followed by transfection with poly(I:C) for 12 h. The mRNA levels of porcine IFN-β, ISG15, ISG54, and ISG56 were determined by qPCR. All experiments were repeated at least three times. Statistical analysis: two-way ANOVA (**A–C**) and two-tailed, unpaired *t*-test (**D–G**).

To validate the antagonistic effect of SVA 3A on IFN-β production, we first compared 3A expression levels in infected and transfected cells by western blotting (Fig. S1C). We then performed luciferase reporter assays in HEK-293T cells. Overexpression of SVA 3A significantly suppressed the Sendai virus (SeV, a model virus widely used to activate RIG-I-mediated interferon responses)-induced IFN-β promoter activity in a dose-dependent manner (Fig. 1A). Similar inhibitory effects were observed for the ISRE and NF-κB promoters (Fig. 1B and C). We then measured endogenous IFN-β expression at both the mRNA and protein levels in the presence of 3A expression in HEK-293T cells; RT-qPCR and ELISA results indicated that 3A overexpression significantly reduced the SeV-induced IFN-β production (Fig. 1D and E). Consistently, the RNA levels of downstream interferon-stimulated genes (ISGs) were also significantly decreased in the context of 3A overexpression (Fig. 1F). We next assessed the effects of SVA 3A on RLR signaling pathways in porcine kidney-15 (PK-15) cells. RT-qPCR data revealed that the 3A overexpression markedly inhibited poly(I:C)-induced expression of IFN-β and ISGs (Fig. 1G). These data indicate that the SVA 3A protein diminishes IFN-β production through targeting the RLR signaling pathway.

### SVA 3A protein interacts with RIG-I to disrupt the RLR signaling pathway

**Fig 2:**
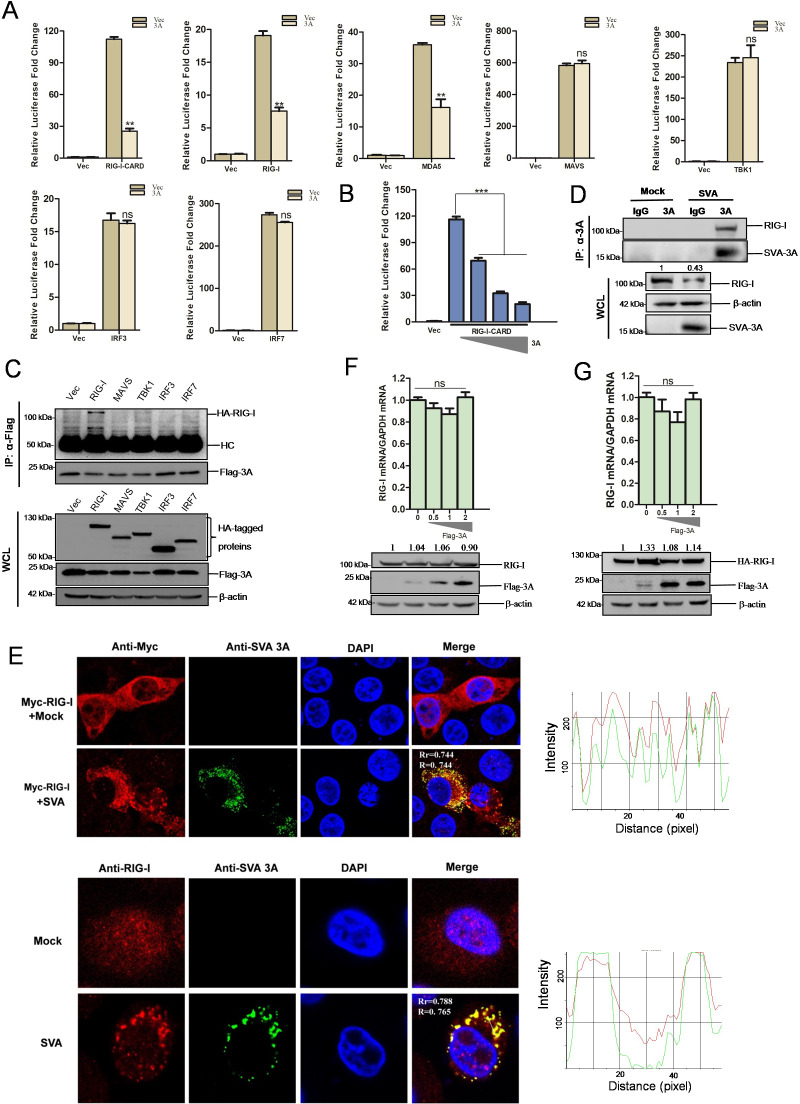
SVA 3A protein interacts with RIG-I. (**A**) HEK-293T cells were co-transfected with plasmids expressing individual RLR signaling pathway molecules expressing plasmids (RIG-I-CARD, RIG-I, MDA5, MAVS, TBK1, IRF3, and IRF7; 100 ng each) and SVA 3A expressing plasmid (100 ng) or empty vector (100 ng), along with ISRE reporter plasmids (50 ng) and PRL-TK control reporter plasmids (5 ng). At 24 h post-transfection, cell lysates were harvested to measure luciferase activity using a dual luciferase assay. (**B**) HEK-293T cells were transfected with increasing doses of SVA 3A expressing plasmid or empty vector (0, 75, 150, or 300 ng) and RIG-I-CARD expressing plasmid (100 ng), together with ISRE reporter plasmids (50 ng) and PRL-TK control reporter plasmids (5 ng) for 24 h. Cell lysates were harvested, and luciferase activity was assessed by dual luciferase assay. (**C**) HEK-293 cells were co-transfected with plasmids expressing RIG-I, MAVS, TBK1, IRF3, or IRF7 and SVA 3A for 24 h. Cell lysates were collected to do an immunoprecipitation assay using an anti-Flag antibody. Immunoprecipitation (IP) samples and whole-cell lysates (WCL) were analyzed by western blotting using the indicated antibodies. (**D**) HEK-293T cells were infected with or without SVA at 1 multiplicity of infection (MOI) for 12 h. Cell lysates were immunoprecipitated with anti-SVA 3A or control IgG antibodies. IP sample and WCL were subjected to western blotting analysis using the indicated antibodies. (**E**) (Upper) PK-15 cells were transfected with Myc-tagged RIG-I plasmids (1 µg) for 24 h, prior to infection with SVA (0.1 MOI) for 12 h. Cells were fixed and permeabilized for immunofluorescence assays using the anti-Myc (red) and anti-SVA 3A (green) Abs to examine the colocalization of exogenous RIG-I and SVA 3A using indicated antibodies. (Lower) PK-15 cells were inoculated with SVA (0.1 MOI) for 12 h. The colocalization of endogenous RIG-I and SVA 3A was observed through immunofluorescence assays using the anti-RIG-I (red) and anti-SVA 3A (green) Abs. Overlapping coefficient (Rr) and Pearson’s correlation (R) values were analyzed using the Image-Pro Plus 6.0 software. (**F**) HEK-293T cells were transfected with SVA 3A plasmid (0, 0.5, 1, and 2 µg) for 24 h. Cell lysates were harvested for qPCR to quantify the levels of RIG-I mRNA and for western blotting analysis to detect the endogenous RIG-I protein levels. (**G**) HEK-293T cells were transfected with HA-tagged RIG-I plasmids (1 µg) and SVA 3A plasmids (0, 0.5, 1, and 2 µg) for 24 h. Similar to panel F, RIG-I mRNA levels were measured using qPCR, and exogenous RIG-I protein levels were detected by western blotting. All experiments were repeated at least three times. Statistical analysis: one-way ANOVA (**B**) and two-tailed, unpaired *t*-test (**A**).

To further determine how SVA 3A disrupts the RLR pathway and impairs the IFN-β production, we assessed its effect on ISRE promoter activation driven by key adapters of the RLRs signaling pathway, including RIG-I-CARD (caspase activation and recruitment domain), RIG-I, MDA5, MAVS, TBK1, IRF3, and IRF7, using luciferase reporter assays in HEK-293T cells. The results showed that the expression of 3A significantly inhibited RIG-I CARD-, RIG-I-, and MDA5-mediated activation of the ISRE promoter but did not interfere with activation induced by other signaling molecules (Fig. 2A). The inhibitory effect of SVA 3A on RIG-I CARD-mediated ISRE promoter activation was observed in a dose-dependent manner (Fig. 2B). We hypothesized that 3A might target molecules upstream of MAVS in the RLR signaling pathway.

We assessed the effects of other SVA nonstructural proteins (L, 2B, 2C, 3C, and 3D) and a host protein, SOCS1, on RIG-I-CARD-induced IFN-β promoter activity. The results suggest that L, 2B, 3C, and 3D also suppressed RIG-I CARD-induced IFN-β promoter activity, whereas 3A exhibited the most potent inhibitory effect, even when expressed at low levels (Fig. S1A). In contrast, 2C and SOCS1 proteins showed no inhibitory effect.

We then performed immunoprecipitation assays to examine the potential interactions between 3A and key molecules (RIG-I, MAVS, TBK1, IRF3, and IRF7) in the RLR signaling pathway. Notably, RIG-I was pulled down by overexpressed 3A (Fig. 2C). Moreover, we confirmed that endogenous RIG-I interacted with 3A in the infection context (Fig. 2D). This interaction was further validated by immunofluorescence assay in both overexpression and infection contexts (Fig. 2E). Previous studies indicated that picornaviral nonstructural proteins can downregulate the expression of host factors to facilitate virus infection (6). We next assessed whether SVA 3A reduces the expression of RIG-I. The results showed the mRNA and protein levels of both endogenous and exogenous RIG-I were unaffected by the presence of SVA 3A (Fig. 2F and G).

**Fig 3:**
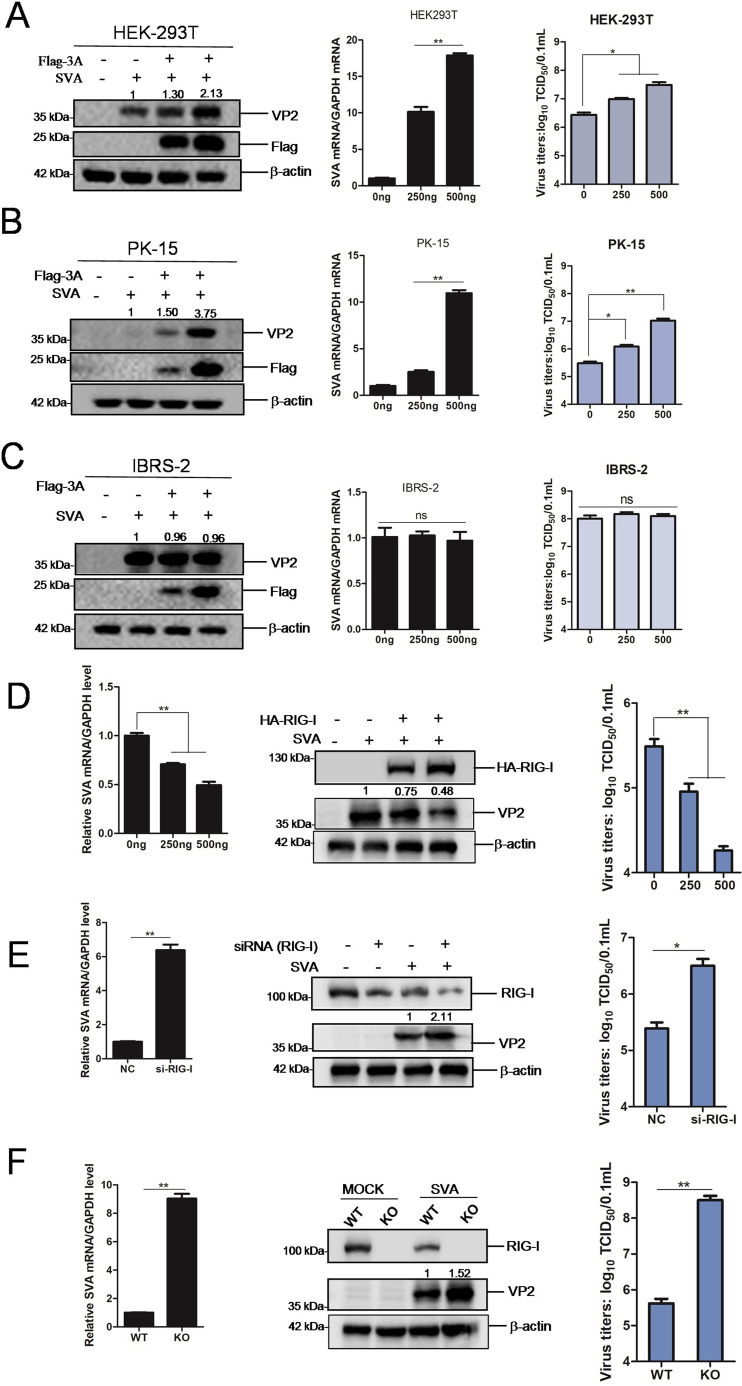
RIG-I inhibits SVA replication. (**A–C**) HEK-293T cells (**A**), PK-15 cells (**B**), or IBRS-2 cells (**C**) were transfected with SVA 3A plasmid (0, 0.25, and 0.5 µg) for 24 h, then inoculated with SVA at 0.1 MOI for 12 h. Cell lysates were collected for quantifying viral mRNA levels by qPCR and for detecting VP2 protein levels by western blotting. Supernatants were harvested to determine viral titers by TCID_50_. (**D**) HEK-293T cells were transfected with HA-tagged RIG-I plasmids (0, 0.25, and 0.5 µg) for 24 h, prior to SVA infection (0.1 MOI) for 12 h. Cell lysates were collected to quantify mRNA and protein levels of SVA, and the supernatants were harvested to determine viral titers by TCID_50_. (**E**) HEK-293T cells were transfected with a siRNA (5′-GAATTTAAAACCAGAATTATC-3′) targeting RIG-I. After 24 h, the cells were infected with SVA at 0.1 MOI for another 12 h. Cells were then harvested to assess the mRNA and protein levels of the virus, and supernatants were harvested to determine viral titers by TCID_50_. (**F**) RIG-I KO and WT PK-15 cells were infected with SVA (0.5 MOI) for 12 h, and the cell lysates were collected. The levels of viral RNA, viral protein, and infectious viral titers were examined. All experiments were repeated at least three times. Statistical analysis: one-way ANOVA (**A–D**), and two-tailed, unpaired *t*-test (**E and F**).

To further investigate whether SVA 3A-mediated disruption of the RLR signaling pathway affects viral replication, we transfected PK-15 and HEK-293T cells, which have an intact RLR signaling pathway, as well as IBRS-2 (Instituto Biologico-Rim Suino-2) with a deficient RLR signaling pathway, with plasmids expressing SVA 3A for 24 h, prior to SVA infection for 12 h. Interestingly, increased levels of viral protein (VP2), viral mRNA, and viral titers were observed in the presence of 3A in HEK-293T (Fig. 3A) and PK-15 cells (Fig. 3B), whereas no effect was observed in IBRS-2 cells (Fig. 3C). Overall, these findings suggest that SVA 3A disrupts RLR signaling pathway transduction through its interaction with RIG-I, which is essential for its antagonistic ability.

Given that SVA 3A targets RIG-I to block activation of the RLR signaling pathway, we examined the role of RIG-I in SVA replication using overexpression (Fig. 3D), knockdown (Fig. 3E), and knockout (Fig. 3F) assays. HEK-293T cells were transfected with plasmids expressing RIG-I for 24 h, followed by SVA infection for 12 h. Overexpression of RIG-I significantly reduced viral titers and the levels of viral protein (VP2) and viral mRNA, indicating an inhibitory effect on SVA replication (Fig. 3D). In contrast, knockdown of RIG-I increased virus titers and the levels of viral protein (VP2) and viral mRNA (Fig. 3E). Consistent with these findings, SVA infection was higher in RIG-I knockout PK-15 cells than in parental wild-type cells (Fig. 3F). These data suggest that RIG-I plays a critical role in restricting SVA replication.

### SVA 3A protein inhibits the formation of the RIG-I/MAVS complex and reduces RIPLET-mediated ubiquitination of RIG-I

**Fig 4:**
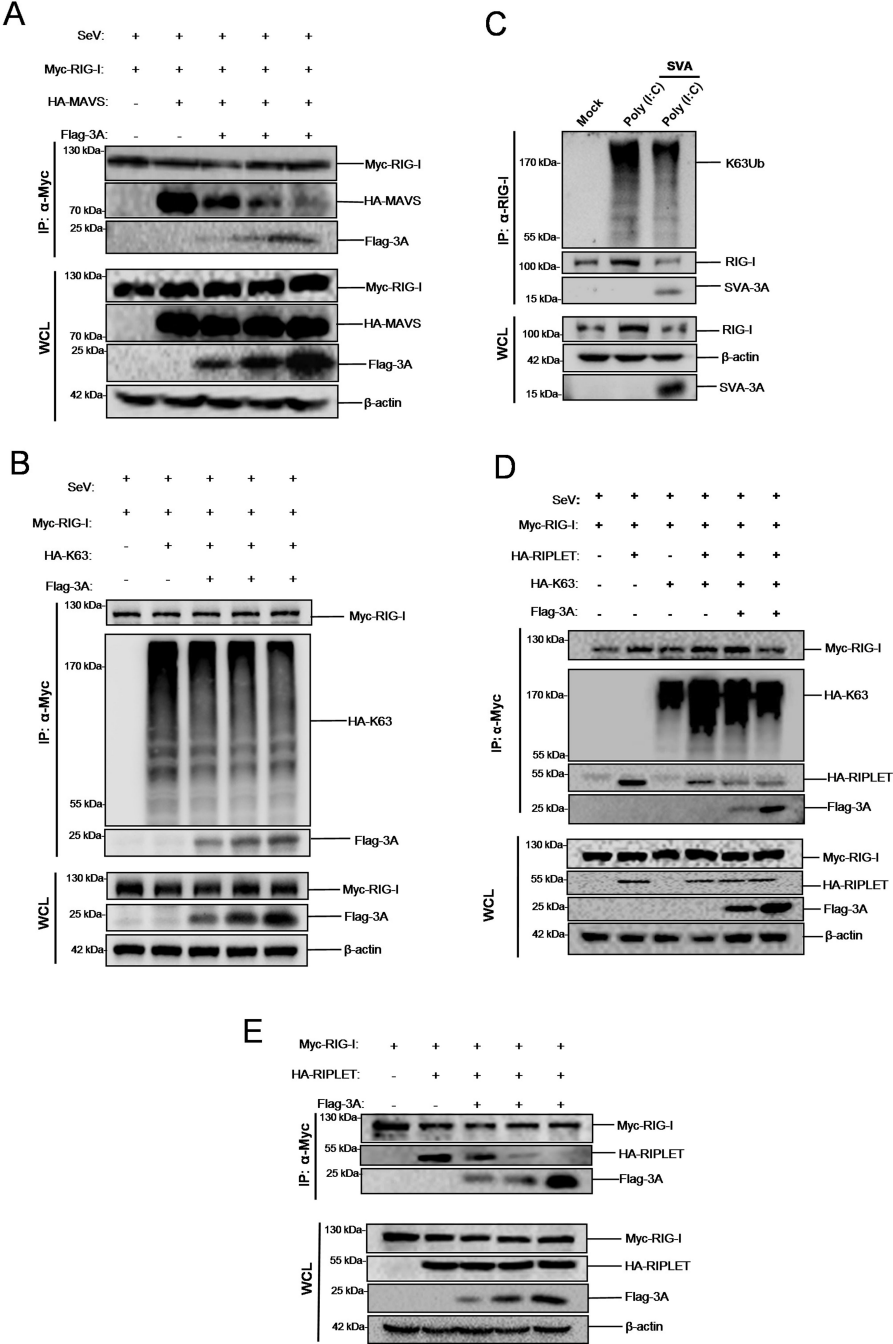
SVA 3A reduces RIPLET-mediated ubiquitination of RIG-I. (**A**) HEK-293T cells were transfected with Myc-RIG-I, HA-MAVS, and increasing amounts of SVA 3A expressing plasmids for 24 h, followed with SeV stimulation for 12 h. Cell lysates were harvested to perform immunoprecipitation using an anti-Myc antibody. Immunoprecipitation (IP) samples and whole-cell lysates (WCL) were analyzed by western blotting with the indicated antibodies. (**B**) HEK-293T cells were transfected with Myc-RIG-I expressing plasmid and HA-tagged K63 ubiquitin plasmids, along with increasing doses of SVA 3A expressing plasmids for 24 h, followed with SeV stimulation for 12 h. Cell lysates were harvested to perform immunoprecipitation using an anti-Myc antibody. IP samples and WCL were analyzed by western blotting to assess the K63-linked ubiquitination of RIG-I. (**C**) PK-15 cells were mock- or poly (I:C)-treated for 6 h, then cells were infected with mock or SVA (0.5 MOI) for another 12 h to evaluate the ubiquitination status of RIG-I in the context of SVA infection. (**D**) HEK-293T cells were co-transfected with the indicated plasmid. At 24 h post-transfection, the transfected cells were treated with SeV for an additional 12 h. Cell lysates were harvested, and an immunoprecipitation assay was performed using anti-Myc antibody. IP samples and WCL were analyzed by western blotting using the indicated antibodies. (**E**) HEK-293T cells were transfected with Myc-RIG-I, HA-RIPLET, and increasing doses of SVA 3A plasmids for 24 h. Cell lysates were collected for immunoprecipitation using anti-Myc antibody. IP samples and WCL were analyzed by western blotting using the indicated antibodies. All experiments were repeated at least three times.

Since the interaction between RIG-I and MAVS is critical for activation of the RLR signaling pathway, we investigated whether SVA 3A affects this interaction. Immunoprecipitation results showed that increasing levels of SVA 3A disrupted the interaction between RIG-I and MAVS in a dose-dependent manner (Fig. 4A). K63-linked ubiquitination of RIG-I is essential for its activation and subsequent interaction with MAVS (20). Because SVA 3A can bind to RIG-I, we next examined whether it affects the K63-linked ubiquitination of RIG-I. To test this, RIG-I and K63-linked ubiquitin were co-expressed with SVA 3A in the presence of SeV stimulation. We observed that the ubiquitination levels of RIG-I gradually decreased as the amount of SVA 3A increased (Fig. 4B). Consistent with the overexpression assay, the levels of poly (I:C)-induced RIG-I ubiquitination were also diminished during SVA infection (Fig. 4C). RIPLET is an E3 ubiquitin ligase that catalyzes K63-linked ubiquitination of RIG-I, which is critical for its activation (21). We therefore examined whether RIPLET-mediated K63-linked ubiquitination was affected by SVA 3A. Although RIPLET can extensively ubiquitinate RIG-I, this ubiquitination was markedly reduced in the presence of SVA 3A (Fig. 4D). Furthermore, we tested whether 3A affects the interaction between RIPLET and RIG-I and found that 3A decreases their interaction in a dose-dependent manner (Fig. 4E). These results reveal that SVA 3A disrupts RIG-I-mediated interferon production by interfering with the interaction between RIPLET and RIG-I, thereby reducing RIPLET-mediated ubiquitination of RIG-I.

### SVA 3A protein binds to the helicase and CARD domains of RIG-I, and amino acids 76–90 are critical for its interferon antagonism

**Fig 5:**
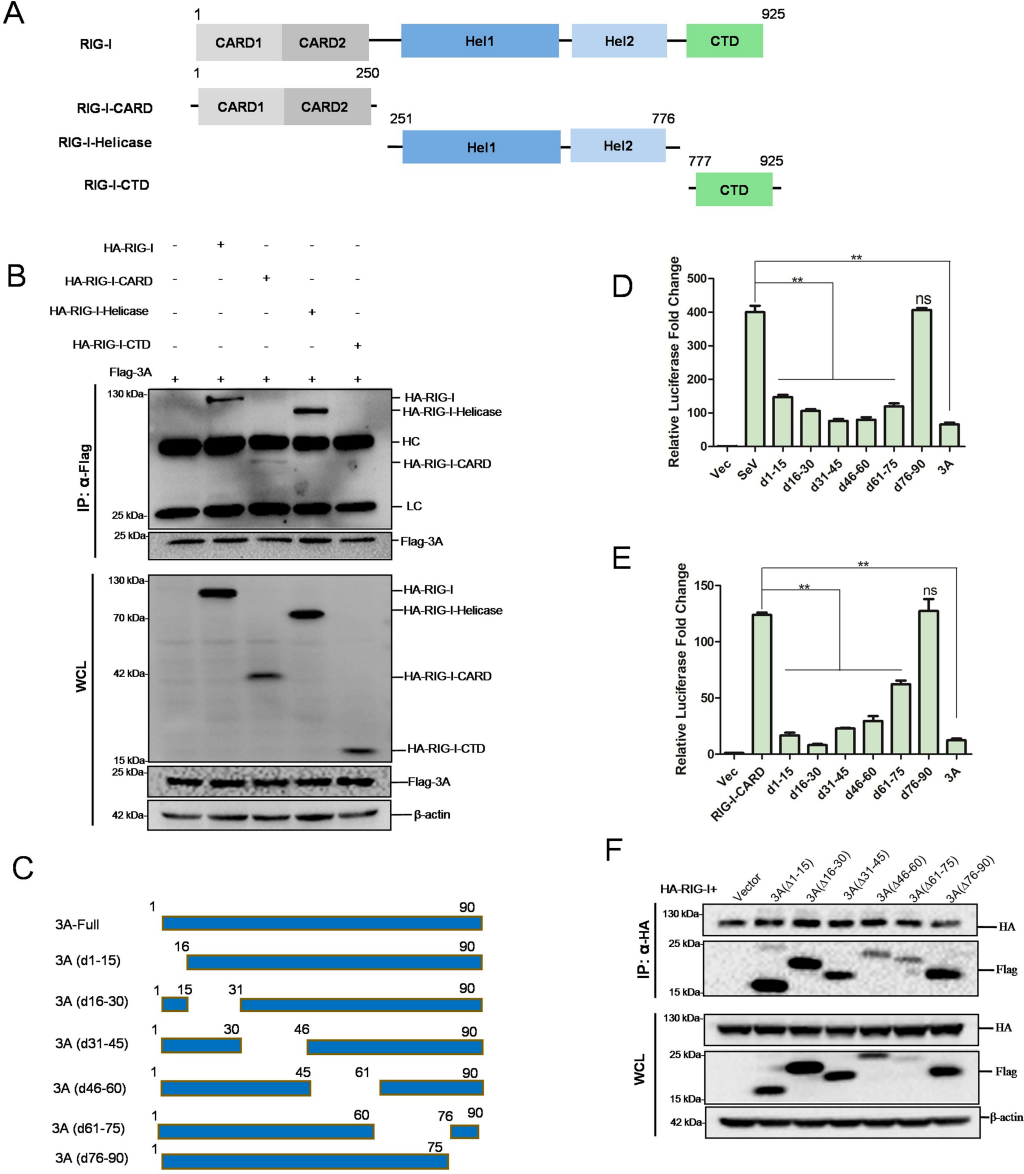
SVA 3A interacts with the helicase and CARD domains of RIG-I. (**A**) Schematic of full-length RIG-I and its truncated mutants. CARD: Caspase Activation and Recruitment Domain; Hel: Helicase Domain; CTD: C-terminal Domain. (**B**) HEK-293T cells were transfected with plasmids expressing HA-tagged full-length RIG-I or its mutants and Flag-tagged SVA 3A expressing plasmids for 24 h. Cell lysates were collected for immunoprecipitation using an anti-Flag antibody. Immunoprecipitation (IP) samples and whole-cell lysates (WCL) were analyzed by western blotting using the indicated antibodies. (**C**) Schematic of full-length SVA 3A and its truncated mutants. (**D**) HEK-293T cells were transfected with the IFN-β reporter plasmids (50 ng), PRL-TK control reporter plasmids (5 ng), and plasmids expressing full-length SVA 3A or indicated truncated mutants for 24 h, followed by treatment with SeV for 12 h. Cell lysates were collected for measuring luciferase activity using dual luciferase assay. (**E**) HEK-293T cells were co-transfected with the ISRE reporter plasmids (50 ng), PRL-TK control reporter plasmids (5 ng), and plasmids expressing full-length SVA 3A or mutant, along with RIG-I CARD expressed plasmids for 24 h. Cell lysates were harvested for dual-luciferase assay. (**F**) HEK-293T cells were transfected with plasmids expressing full-length SVA 3A or truncated mutant and HA-RIG-I for 24 h. Cell lysates were harvested for immunoprecipitation. All experiments were repeated at least three times. Statistical analysis: one-way ANOVA (**D, E**).

RIG-I comprises three major domains: the CARD domains, the helicase domain, and the CTD domain (Fig. 5A). To identify which domain of RIG-I interacts with the SVA 3A protein, we constructed plasmids expressing the CARDs, helicase, or CTD domains and then performed an immunoprecipitation assay. HEK-293T cells were transfected with plasmids expressing SVA 3A together with CARD, helicase, or CTD domain for 24 h. The results showed that SVA 3A was able to interact with both the helicase and CARD domains, suggesting that multiple binding sites are present in RIG-I (Fig. 5B).

To identify the key functional region of SVA 3A responsible for dysregulating RLR signaling pathway, we constructed a series of 3A truncated mutants (Δ amino acid [aa] 1–15, Δ aa 16–30, Δ aa 31–45, Δ aa 46–60, Δ aa 61–75, and Δ aa 76–90) (Fig. 5C). The activation status of SeV-triggered IFN-β promoter and the RIG-I-CARD-mediated ISRE promoter was analyzed in the presence of these mutants, respectively. The results showed that the deletion of the aa 76–90 region of the 3A protein significantly abrogated its suppressive effects on SeV-induced IFN-β promoter activation and RIG-I-CARD-induced ISRE promoter activation (Fig. 5D and E). In addition, the interactions between the SVA 3A mutants and RIG-I were examined; all mutants interacted with RIG-I, indicating multiple binding sites are also present within the 3A protein (Fig. 5F). To validate our findings in the context of infection, we attempted to construct a recombinant SVA lacking amino acids 76–99 in the 3A region. However, we were unable to rescue the recombinant virus, suggesting that this region is critical for SVA replication and that its deletion may be lethal to the virus.

To further determine the key residues within aa 76–90 of the SVA 3A protein that are responsible for its antagonistic effect on RIG-I, we first generated three 3A mutants lacking aa 76–80, 81–85, or 86–90 (d76–80, d81–85, and d86–90) and assessed their ability to inhibit IFN-β promoter activity induced by RIG-I-CARD. All three mutants lost their antagonistic effects on the RLR pathway (Fig. S2A). We next generated another set of 3A mutants in which residues at aa 76–80, 81–85, or 86–90 were replaced with alanines (M76–80, M81–85, M86–90, and M76–90) and tested their effects on IFN-β promoter activity and interaction with RIG-I. These mutants lost their inhibitory effects on the RLR signaling pathway (Fig. S2B) but still can bind to RIG-I (Fig. S2C). These results indicate that multiple critical residues likely exist within aa 76–90 and that this region of 3A is essential for its interferon antagonism.

We then predicted the SVA 3A-RIG-I complex using UCSF ChimeraX and analyzed the interaction interface to identify potential contact residues. Several binding sites were predicted between 3A and RIG-I (Fig. S2D). To functionally validate the roles of these amino acids in 3A activity, we selected four amino acids with high interaction scores, K85 and T78 within the region of aa 76–90, and D72 and K66 outside this region. Four single 3A mutants (K85A, T78A, D72A, or K66A), a quadruple mutant containing four amino acid substitutions (K85A/T78A/D72A/K66A), and a double mutant containing two amino acid substitutions (K85A/T78A) within the aa 76–90 region were constructed and evaluated for their inhibitory effects on IFN-β promoter activation. None of these mutations can significantly affect the ability of 3A to inhibit the RLR signaling pathway (Fig. S2E). We also performed an immunoprecipitation assay to test the effects of these mutations on the interaction between RIG-I and 3A and found that none of them altered the interaction between SVA 3A and RIG-I (Fig. S2F).

### 3A proteins from other picornaviruses dysregulate RIG-I activation

**Fig 6:**
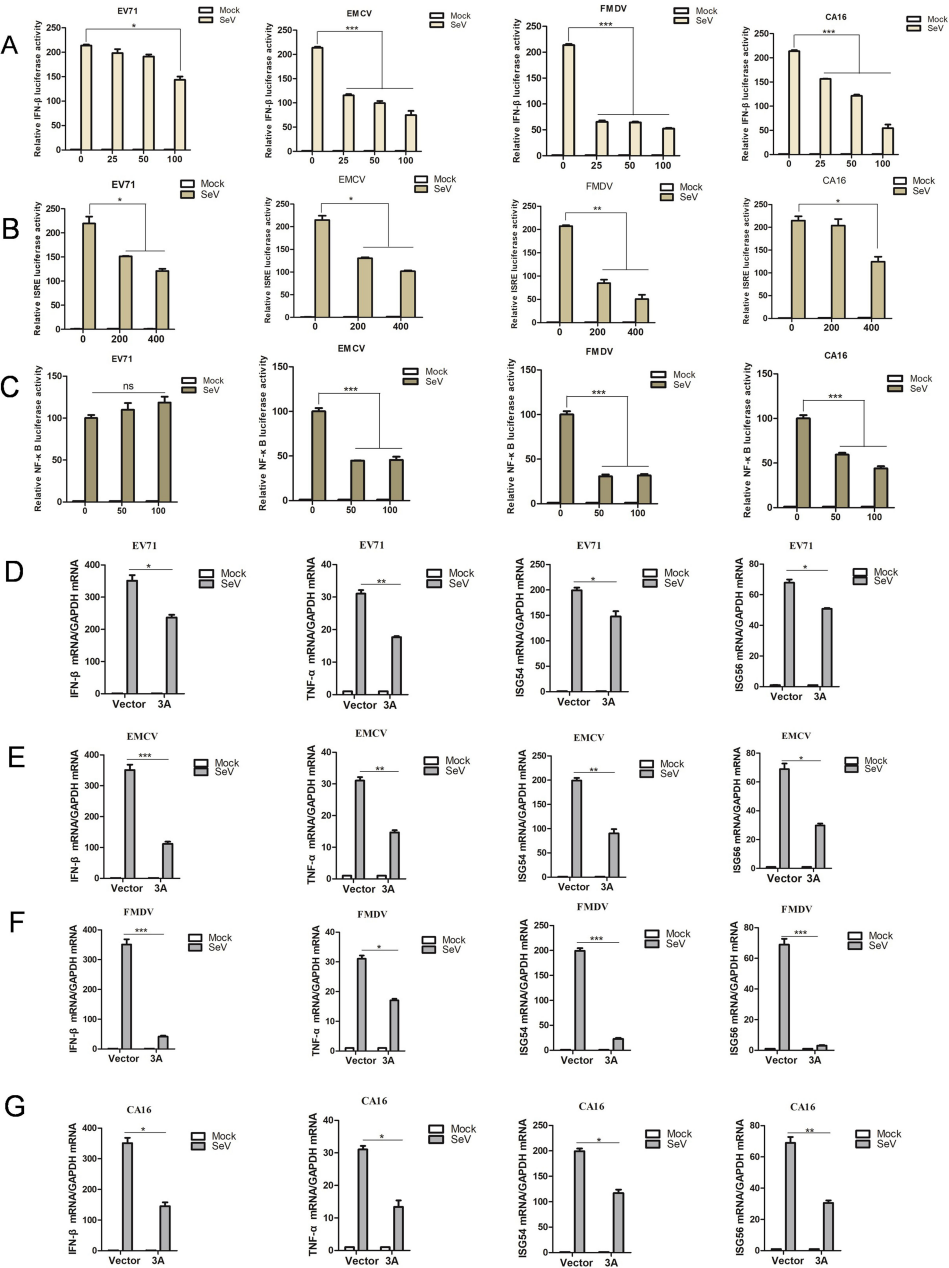
The 3A proteins of EMCV, CA16, EV71, and FMDV suppress the RLR pathway transduction. (**A–C**) HEK-293T cells were transfected with the indicated reporter plasmids (50 ng each): IFN-β (**A**), ISRE (**B**), and NF-κB (**C**), PRL-TK control reporter (5 ng), and increased doses of plasmids expressing the indicated picornaviral 3A for 24 h, followed by treatment with SeV for 12 h. Cell lysates were collected for measuring luciferase activity using a dual luciferase assay. (**E–G**) HEK-293T cells were transfected with the indicated picornaviral 3A: EV71 (**D**), EMCV (**E**), FMDV (**F**), and CA16 (**G**) for 24 h, followed by SeV stimulation for 12 h. Cell lysates were collected to evaluate the mRNA levels of the indicated ISGs. All experiments were repeated at least three times. Statistical analysis: two-way ANOVA (**A–C**) and two-tailed, unpaired *t*-test (**D–G**).

The above findings prompted us to further investigate whether 3A from other picornaviruses also affects RIG-I’s functions. We selected several representative viruses from different genera, including EMCV, CA16, and EV71. Because we previously found that FMDV 3A interacted with RIG-I and downregulated its expression at the mRNA levels to antagonize the RLR signaling pathway (12), we included it as a control. We first assessed the effect of these 3A proteins on SeV-induced activation of the RLR signaling pathway. The 3A proteins of EMCV, FMDV, and CA16 could reduce the promoter activity of IFN-β, ISRE, and NF-κB, whereas EV71 3A decreased the promoter activities of IFN-β and ISRE but did not affect NF-κB (Fig. 6A through C). The mRNA levels of IFN-β, TNF-α, ISG54, and ISG56 were further assessed in the presence of these picornavirus 3A proteins, and the results revealed that all these 3A proteins significantly suppressed SeV-induced expression of IFN-β, TNF-α, ISG54, and ISG56 (Fig. 6D through G).

**Fig 7:**
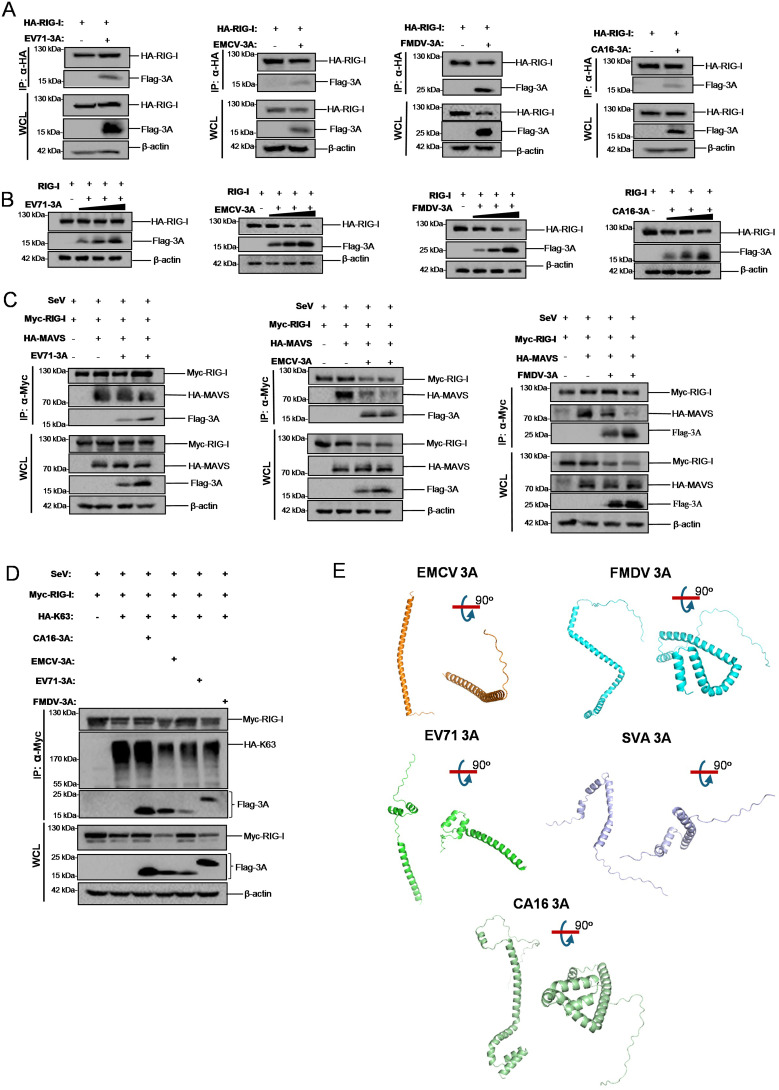
The impacts of picornaviral 3A on RIG-I activity. (**A**) HEK-293T cells were transfected with an HA-RIG-I expressing plasmid and the indicated picornaviral 3A expressing plasmids. After 24 h, cell lysates were subjected to immunoprecipitation using anti-HA antibody and then analyzed by western blotting to evaluate the interaction. (**B**) HEK-293T cells were transfected with HA-RIG-I expressing the plasmid and the indicated picornaviral 3A expressing plasmids. After 24 h, cell lysates were analyzed by western blotting to evaluate the protein expression. (**C**) HEK-293T cells were transfected with a plasmid expressing Myc-RIG-I and HA-MAVS, together with increased amounts of the indicated picornaviral 3A expressing plasmids for 24 h, followed with SeV stimulation for another 12 h. Cell lysates were subjected to immunoprecipitation using anti-Myc antibody and then analyzed by western blotting to evaluate the interaction between RIG-I and MAVS. (**D**) HEK-293T cells were transfected with Myc-RIG-I expressing plasmid and HA-tagged K63 ubiquitin plasmids, along with the indicated picornaviral 3A expressing plasmids for 24 h. Then, the transfected cells were treated with SeV for an additional 12 h, and cell lysates were assessed to immunoprecipitation using anti-Myc antibody to detect the K63-linked ubiquitination of RIG-I. (**E**) The structure of picornaviral 3A was predicted using ColabFold. GenBank accession numbers were JQ864080.1 (EMCV), GU931682.1 (FMDV), KY747510 (SVA), KC342228.1 (CA16), and GU396280.1 (EV71). All experiments were repeated at least three times.

We then examined whether these 3A proteins also affect RIG-I through degradation, interaction, or reduced ubiquitination. We observed that: (i) all of these 3A proteins interacted with RIG-I (Fig. 7A), (ii) EMCV and FMDV 3A reduced RIG-I expression (Fig. 7B), and (iii) EV71, EMCV, and FMDV 3A reduced the interaction between RIG-I and MAVS and decreased the levels of K63-linked ubiquitination of RIG-I (Fig. 7C and D). However, we did not determine how CA16 3A influences RIG-I activation after the binding. Given that 3A proteins of picornaviruses vary in their amino acid sequence, we predicted the three-dimensional structures of these 3A proteins (Fig. 7E). Despite having low amino acid sequence identity, they all contain a large α-helix at the N-terminal, followed by a short C-terminal tail (Fig. 7E and Fig. S1B). We suggest that structure is likely more important than amino acid sequence in determining the interaction with RIG-I. Collectively, these findings show that in addition to SVA 3A, 3A proteins from other picornaviruses also act as interferon antagonistic proteins by inhibiting the RLR signaling pathway through targeting RIG-I.

## DISCUSSION

Our data reveal that picornavirus 3A proteins act as interferon antagonists by disrupting the activation of the RLR signaling pathway by targeting RIG-I, a critical RNA sensor. We found that SVA 3A interacts with RIG-I by binding to its CARD and helicase domains; this interaction reduced RIPLET-mediated ubiquitination of RIG-I, thereby suppressing its activation and interfering with the formation of the RIG-I and MAVS complex, ultimately antagonizing the host antiviral response by impairing downstream signaling. Intriguingly, a similar phenomenon was also observed for 3A proteins from other picornaviruses, including EV71, EMCV, FMDV, and CA16, which likewise target RIG-I to suppress interferon production. However, the mechanisms by which these viral 3A proteins affect RIG-I activity differ to some extent. All of these 3A proteins can bind to RIG-I. EMCV and FMDV 3A reduce both the expression and ubiquitination of RIG-I, whereas EV71 3A reduces ubiquitination without affecting expression, and CA16 3A affects neither. Previous studies have reported that different picornaviruses utilize various strategies to modulate RIG-I activation and evade innate immune response (6, 22–27). Here, our findings further suggest that targeting RIG-I through 3A protein is a common strategy used by different picornaviruses.

Initially, MDA5 was thought to be the primary sensor of viral RNA during picornavirus replication, triggering the host antiviral defense response (28). *In vivo* studies have shown that MDA5 knockout mice are more susceptible to EMCV infection compared to RIG-I knockout mice (29), suggesting that MDA5 plays a dominant role in recognizing picornavirus RNA and activating the innate immune response. It is therefore reasonable to speculate that MDA5 may be dysregulated during picornavirus infection, and numerous studies have supported this point (6). However, it remains puzzling why picornaviruses specifically antagonize RIG-I, given that RIG-I was not originally thought to sense picornaviral RNA. Several picornaviral proteins, such as 3C^pro^, L^pro^, 2B, and 3B, have been reported to modulate RIG-I activity via various mechanisms (22, 24, 26, 27, 30). In this study, we found RIG-I can also be targeted by 3A proteins from different picornaviruses. These findings imply that RIG-I might play an important role in the host antiviral response against picornavirus. Interestingly, coronaviruses were also originally thought not to be sensed by RIG-I, but recent studies revealed that RIG-I can recognize SARS-CoV-2 RNA genome to induce an antiviral response (31). Our recent study also demonstrated that RIG-I is critical for inducing interferon production during SVA infection (5). In addition, RIG-I can be upregulated as an interferon-stimulated gene (ISGs) during viral infection (32). The increased expression of RIG-I may further amplify antiviral signaling cascades and help restrict viral infection (1). Further studies should focus on identifying potential motifs within picornaviral genomes that are recognized by RIG-I. For instance, the poly-U/UC region within the 3′ untranslated region of the HCV RNA genome has been shown to bind to RIG-I, enhancing its activation (33).

**Fig 8:**
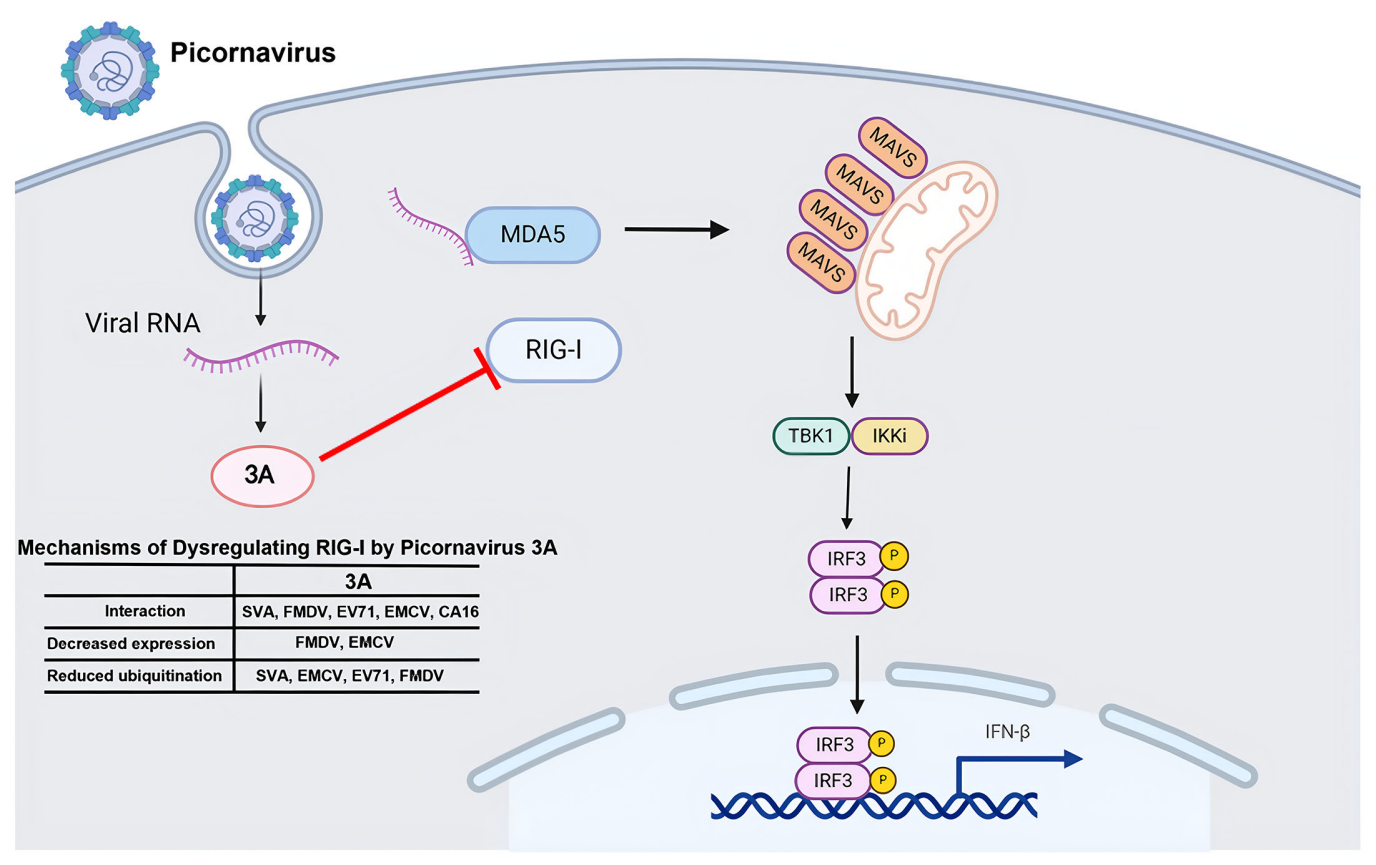
Proposed models of the picornavirus 3A protein antagonizing the RLR signaling pathway by dysregulating RIG-I. When picornaviruses infect host cells, its genomic RNA is translated into a single polyprotein that is subsequently cleaved by viral proteases into nonstructural and structural proteins. Among these, the 3A protein contributes to the remodeling of host intracellular membranes to form replication organelles for viral RNA synthesis. At the same time, 3A also acts as an interferon antagonist by dysregulating RIG-I activity and impairing its ability to sense viral RNA, thereby suppressing activation of the host antiviral immune response. Picornaviral 3A employs similar yet distinct mechanisms to dysregulate RIG-I, including direct interaction, downregulation of its expression, and reduction of its ubiquitination.

Although the amino acid sequences of 3A proteins are divergent within the *Picornaviridae* family (8)*,* their structures are highly conserved, featuring a large N-terminal and a short C-terminal tail; this highly similar structure suggests that the functions of 3A protein during the picornaviral lifecycle may also be conserved. Picornaviral 3A is critical for the formation of the viral replication organelle (RO), a subcellular membrane-bound structure that serves as a platform for viral RNA synthesis (8, 34–36). The RO is essential for viral infection, enhancing RNA synthesis efficiency, coordinating viral replication processes, and shielding viral RNA from detection by cytoplasmic innate immune sensors (37, 38). Previous studies have shown that the viral nonstructural proteins essential for RO formation, including Coronavirus nsp3 and Flavivirus NS4A (39–41), can also impair the RLR signaling pathway and suppress interferon production to promote viral infection (42–44). Based on these findings, we propose a model in which picornaviral 3A protein facilitates efficient infection through dual functions (Fig. 8). When a picornavirus infects host cells, 3A remodels intracellular membranes to form replication organelles for viral RNA synthesis. Meanwhile, 3A targets RIG-I and suppresses its activation to enable the virus to evade the host antiviral response. Recent cryo-electron tomography studies have shown that coronaviruses nsp3 and nsp4 are the essential viral components of the double-membrane vesicles (DMVs) (39, 40), highlighting the central role of these viral membrane remodeling proteins in protecting viral RNA from innate immune recognition. An important question for future investigation is whether 3A itself is a core viral factor required for RO formation, which would provide further insight into its multifunctional roles in viral replication and immune evasion.

We acknowledge several limitations of this study. (i) We were unable to rescue a recombinant SVA lacking aa 76–90, which limited our ability to investigate the effect of 3A on RIG-I in the infection context. Whether the analogous region (aa 76–90) in other picornaviruses is indispensable for viral replication remains to be determined. In addition, generating recombinant viruses lacking all or partial 3A may help further understand the interplay between 3A and RIG-I during infection. (ii) Although we generated truncated and alanine-substitution mutants of 3A within aa 76–90, all of these mutants lost their ability to antagonize the RLR signaling pathway but still retained interaction with RIG-I, suggesting that multiple residues may collectively contribute to the antagonistic activity of 3A. Additional structural and biochemical analyses are needed to define the detailed binding interface between 3A and RIG-I, which may inform the generation of recombinant SVA mutants to further dissect the interplay between 3A and RIG-I in the context of infection.

In summary, our findings show that picornaviral 3A proteins function as antagonists for interferon production by specifically targeting RIG-I, a critical RNA virus sensor in the RLR pathway. Of note, although all of these 3A proteins bind to RIG-I, they dysregulate RIG-I activity through partially distinct mechanisms. These findings expand our knowledge of interferon antagonistic strategies employed by picornaviruses.

## MATERIALS AND METHODS

### Cell and virus

Human embryonic kidney 293T (HEK-293T) cells and porcine kidney PK-15 were maintained in Dulbecco’s modified Eagle medium (DMEM, Invitrogen) supplemented with 10% fetal bovine serum (FBS) and 100 U/mL penicillin-streptomycin, and cultured in a humidified incubator at 37°C with 5% CO_2_. SVA strain CH-FJ-2017 (GenBank accession number KY747510) was used in the present study. SeV was kindly provided by Prof. Hongbing Shu (Wuhan University, China).

### Plasmids and reagents

The cDNAs of RIPLET and K63 ubiquitin with HA-tag were synthesized and cloned into the pcDNA3.1 vector. Viral 3A cDNA (EV71, FMDV, SVA, and EMCV) was amplified from the viral genome and cloned into the p3xFLAG-CMV-7.1 vector, and 3A of CA16 was synthesized and cloned into the p3xFLAG-CMV-7.1 vector. Plasmids expressing HA-RIG-I mutants (CARD, helicase, and CTD) were constructed using the PCR-based mutagenesis, as previously described (45). The IFN-stimulated response element (ISRE), IFN-β, NF-kB promoter luciferase reporter plasmids, pRL-TK internal control plasmids, and HA-tagged MAVS-, TBK1-, IRF3-, and IRF7-expressing plasmids were kind gifts from Dr. Hongbing Shu (Wuhan University, China).

Antibodies and other reagents used in this study include anti-Flag, anti-HA, and anti-Myc mouse mAb (Sigma-Aldrich), anti-β-Actin mouse Ab (Proteintech), HRP-goat anti-Rabbit and mouse (H + L) (BIODRAGON), goat anti-Mouse IgG (H + L) Cross-Adsorbed Secondary Antibody with Alexa Fluor 594 (Thermo Fisher Scientific), Goat anti-Rabbit IgG (H + L) Cross-Adsorbed Secondary Antibody with Alexa Fluor 488 (Thermo Fisher Scientific), IFN-beta ELISA (Raybiotech), and jetPRIME kit (Polyplus Transfection). Anti-SVA 3A and SVA VP2 rabbit polyclonal Abs were made in-house.

### Dual-luciferase reporter assay

HEK-293T cells (5 × 10^4^/well) were seeded in 24-well plates and transfected with plasmids expressing the indicated reporter (50 ng) and pRL-TK control reporter (5 ng), and plasmids expressing the viral protein using jetPRIME DNA transfection reagent for the specified duration. SeV was used in the indicated experiments. Cells were lysed in 1× passive lysis buffer, and luciferase activity was measured following the manufacturer’s protocol (Promega). Relative firefly luciferase activity, normalized to the Renilla luciferase activity, was used to represent the data.

### Co-immunoprecipitation and western blotting

HEK-293T cells plated in 100 mm dishes were transfected with the indicated plasmids for 24 h before being lysed in the ice-cold NP-40 lysis buffer for immunoprecipitation, as described previously (46). Briefly, the cell lysates were immunoprecipitated with 50% (vol/vol) slurry of GammaBind G Plus-Sepharose (GE Health Care Life Sciences), binding with the indicated antibodies at 4°C overnight. Protein samples were mixed with SDS loading buffer and processed for western blotting analysis. Supernatants were electrophoresed in 10% polyacrylamide gels and transferred onto BioTraceTM NT nitrocellulose membrane, followed by immunoblotting with the indicated antibodies.

### Immunofluorescence focus assay

PK-15 cells plated in a 30 mm cell culture dish with a glass bottom were transfected with or without Myc-RIG-I for 24 h, prior to infection with SVA for 12 h at 0.1 MOI. All cells were fixed with 4% paraformaldehyde in PBS and then overlaid with 0.2% Triton X-100 for permeabilization. The cells were incubated with the indicated antibody, diluted in blocking buffer (5% BSA), at room temperature. Stained cells were examined and imaged with a Nikon eclipse 80i fluorescence microscope and NIS Elements F 2.30 software.

### RNA extraction and real-time qPCR

Total RNAs from cells were extracted using Trizol reagent (moibio) according to the manufacturer’s protocol. Reverse transcription was performed with HiScript II Q RT SuperMix for qPCR (Vazyme) to synthesize complementary DNA (cDNA). The cDNAs were used to analyze the target genes of interest using ChamQ Universal SYBR qPCR Master Mix (Vazyme) and gene-specific primers. Glyceraldehyde-3-phosphate dehydrogenase (GAPDH) was used as a housekeeping gene for normalization. The relative transcript levels of the indicated genes were calculated using the 2^-ΔΔCT^ method. All qPCR primers are listed in Table S1.

### Statistical analysis

All data are represented as mean ± SEM from at least three independent experiments. Statistical analysis using one-way ANOVA test and unpaired *t*-test is described in the figure legends. *P* < 0.05 was considered to be statistically significant. *P < 0.05, **P < 0.01, ***P < 0.001; ns, not significant.

## Data Availability

All data supporting this study are included in the article.

## SUPPLEMENTAL MATERIAL

Supplemental material jvi.02069-25-s0001.pdf Fig. S1 and S2; Table S1.

ASM does not own the copyrights to Supplemental Material that may be linked to, or accessed through, an article. The authors have granted ASM a non-exclusive, world-wide license to publish the Supplemental Material files. Please contact the corresponding author directly for reuse.
