# Supplementary material for: Dysregulation of RIG-I activation by picornavirus 3A protein
Source: J Virol. 2026 May 20;100(6):e02069-25. doi: 10.1128/jvi.02069-25 (PMC13288468; doi:10.1128/jvi.02069-25)
Supplement: Supplemental material — Fig. S1 and S2; Table S1. [file jvi.02069-25-s0001.pdf]

Supplementary Materials

Dysregulation of RIG-I activation by picornavirus 3A protein

Xiangle Zhang[1,#], Zhenxiang Zhao[1,#], Kangli Li[1,#], Wenzhe Chen[1], Guanshun Wang[1], Fan Yang[1], Guoliang Zhu[1], Jijun He[1], Xi Lan[1], Haixue Zheng[1], Pengfei Li[2], Zixiang Zhu[1]

1. State Key Laboratory of Veterinary Etiological Biology, College of Veterinary Medicine, Lanzhou University, Lanzhou Veterinary Research Institute, Chinese Academy of Agricultural Sciences, Lanzhou, China

2. Department of Medicine, Washington University School of Medicine, St. Louis, MO, USA

Corresponding author:

Address Correspondence to Zixiang Zhu, zhuzixiang@caas.cn; Haixue Zheng, zhenghaixue@caas.cn; Pengfei Li, lipengfei231x@foxmail.com.

# contributed equally to this article.

This PDF files includes:

Supplementary figures legends

Supplementary figures S1-S2

Supplementary Table 1

Figure S1. The role of SVA nonstructural proteins in RIG-I-CARD mediated IFN-β promoter activity.

(A) HEK-293T cells were transfected with IFN-β reporter plasmids (50 ng), internal control plasmid PRL-TK (5 ng), RIG-I-CARD plasmid (50 ng), and empty vector (Vec) or the indicated plasmids expressing viral proteins or a host SOCS1 protein for 24 h. Cell lysates were harvested for luciferase activity measured by dual luciferase assay. (B) The alignment of 3A protein sequences from different picornaviruses. (C) HEK-293T cells were infected with SVA (0, 1, 5 or 10 MOI) for 12 h, or transfected with SVA 3A expressing plasmids (0, 125, 250 or 500 ng) for 24 h. Cell lysates were collected for Western blotting analysis using an anti-SVA 3A antibody.

Figure S2. Identification of the crucial region between the interaction of RIG-I and SVA 3A.

(A) HEK-293T cells were transfected with IFN-β reporter plasmids (50 ng), internal control plasmid PRL-TK (5 ng), RIG-I-CARD plasmid (50 ng), and empty vector (Vec), or full length or the deletion mutations of 3A protein-expressing plasmids (50 ng) for 24 h. Cell lysates were harvested for luciferase activity measured by dual luciferase assay. (B) HEK-293T cells were transfected with IFN-β reporter plasmids (50 ng), internal control plasmid PRL-TK (5 ng), RIG-I-CARD plasmid (50ng), and empty vector (Vec), or plasmid expressing indicated 3A mutants (50 ng) for 24 h. Cell lysates were harvested for luciferase activity measured by dual luciferase assay and for protein expression detection by Western blotting. (C) HEK-293T cells were transfected with HA-RIG-I (1 μg) and the indicated 3A mutant (1 μg) for 24 h. Cell lysates were collected for immunoprecipitation assay using anti-Flag antibody. (D) The interaction sites between SVA 3A and RIG-I was predicted using UCSF ChimeraX software. (E) HEK-293T cells were transfected with IFN-β reporter plasmids (50 ng), internal control plasmid PRL-TK (5 ng), RIG-I-CARD plasmid (50ng), and empty vector (Vec), or indicated 3A mutants (50 ng) for 24 h. Cell lysates were harvested for luciferase activity measured by dual luciferase assay and for protein expression detection by Western blotting. (F) HEK-293T cells were transfected with HA-RIG-I (1 μg) and the 3A mutant-expressing plasmid (1 μg) for 24 h. Cell lysates were collected for immunoprecipitation assay using anti-Flag antibody.

Fig.S1

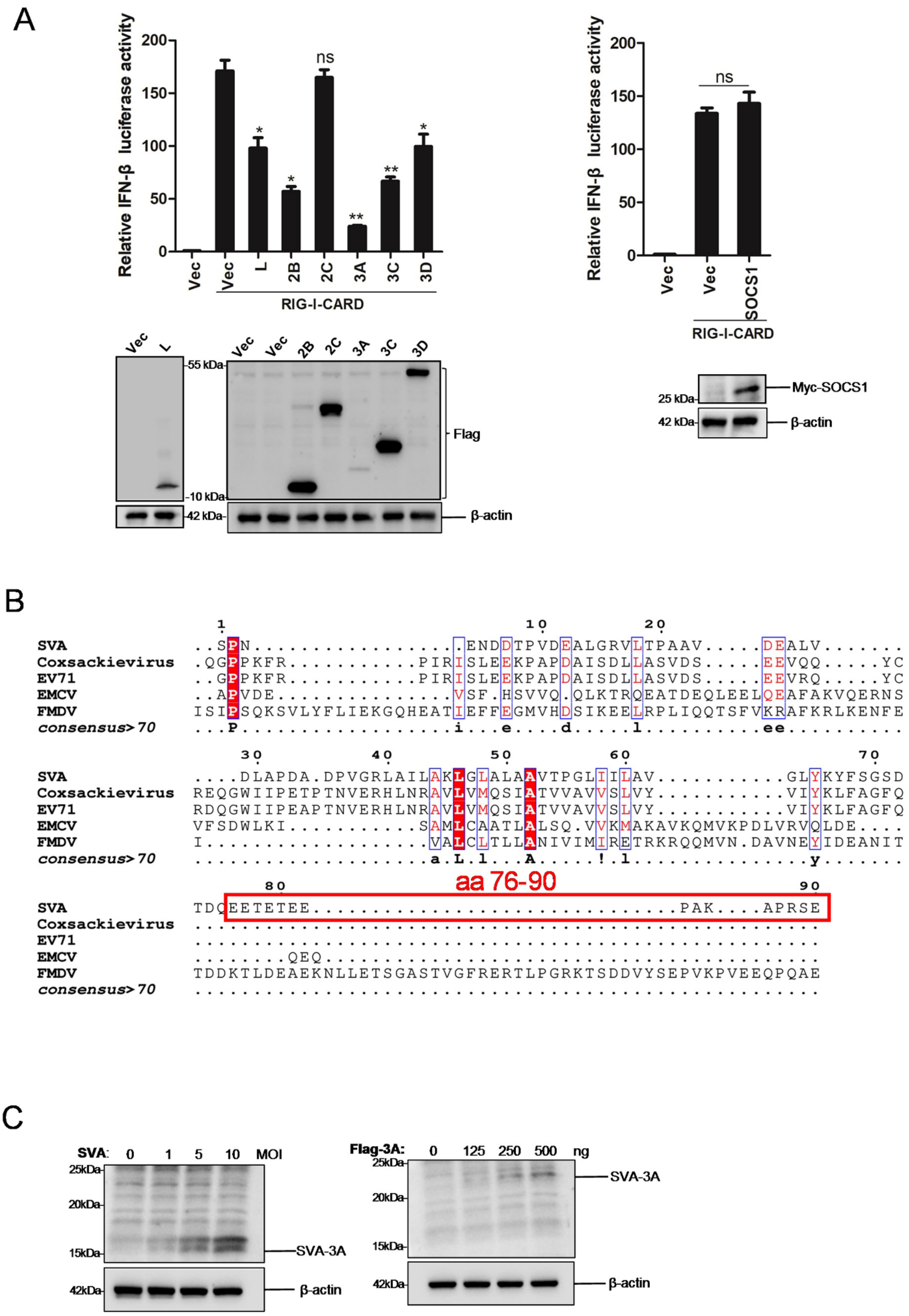
A
Relative IFN-β luciferase activity
200
150
100
50
0
ns
*
**
Vec
L
2B
2C
3A
3C
3D
RIG-I-CARD
SOCS1
55 kDa
10 kDa
42 kDa
Flag
β-actin
Myc-SOCS1
25 kDa
B
SVA
Coxsackievirus
EV71
EMCV
FMDV
consensus>70
..SPN.................ENDDTPVDEALGRVLTPAAV.....DEALV.......
.QGPPKFR...........PIRISLEEKPAPDAISDLLASVDS.....EEVQQ.....YC
..GPPKFR...........PIRISLEEKPAPDAISDLLASVDS.....EEVRQ.....YC
..APVDE...............VSF.HSVVQ.QLKTRQEATDEQLEELQEAFAKVQERNS
ISIPSQKSVLYFLIEKGQHEATIEFFEGMVHDSIKEELRPLIQQTSFVKRAFKRLKENFE
...P..................i...e...d.....l..........ee.........
....DLAPDA.DPVGRLAILAKLGLALAAVTPGLIILAV...........GLYKYFSGSD
REQGWIIPETPTNVERHLNRAVLVMQSIATVVAVVSLVY..........VIYKLFAGFQ
RDQGWIIPEAPTNVERHLNRAVLVMQSIATVVAVVSLVY..........VIYKLFAGFQ
VFSDWLKI..........SAMLCAATLALSQ.VVKMAKAVKQMVKPDLVRVQLDE....
I.................VALCLTLLANIVIMIRETRKRQQMVN.DAVNEYIDEANIT
..................a.L.l...A.....!.l...............y.......
aa 76-90
TDQEETETEE............................PAK....APRSE
....
....
........QEQ..
TDDKTLDEAEKNLLETSGASTVGFRERTLPGRKTSDDVYSEPVKPVEEQPQAE
....
C
SVA: 0 1 5 10 MOI
25kDa
20kDa
15kDa
42kDa
SVA-3A
β-actin
Flag-3A: 0 125 250 500 ng

Fig.S2

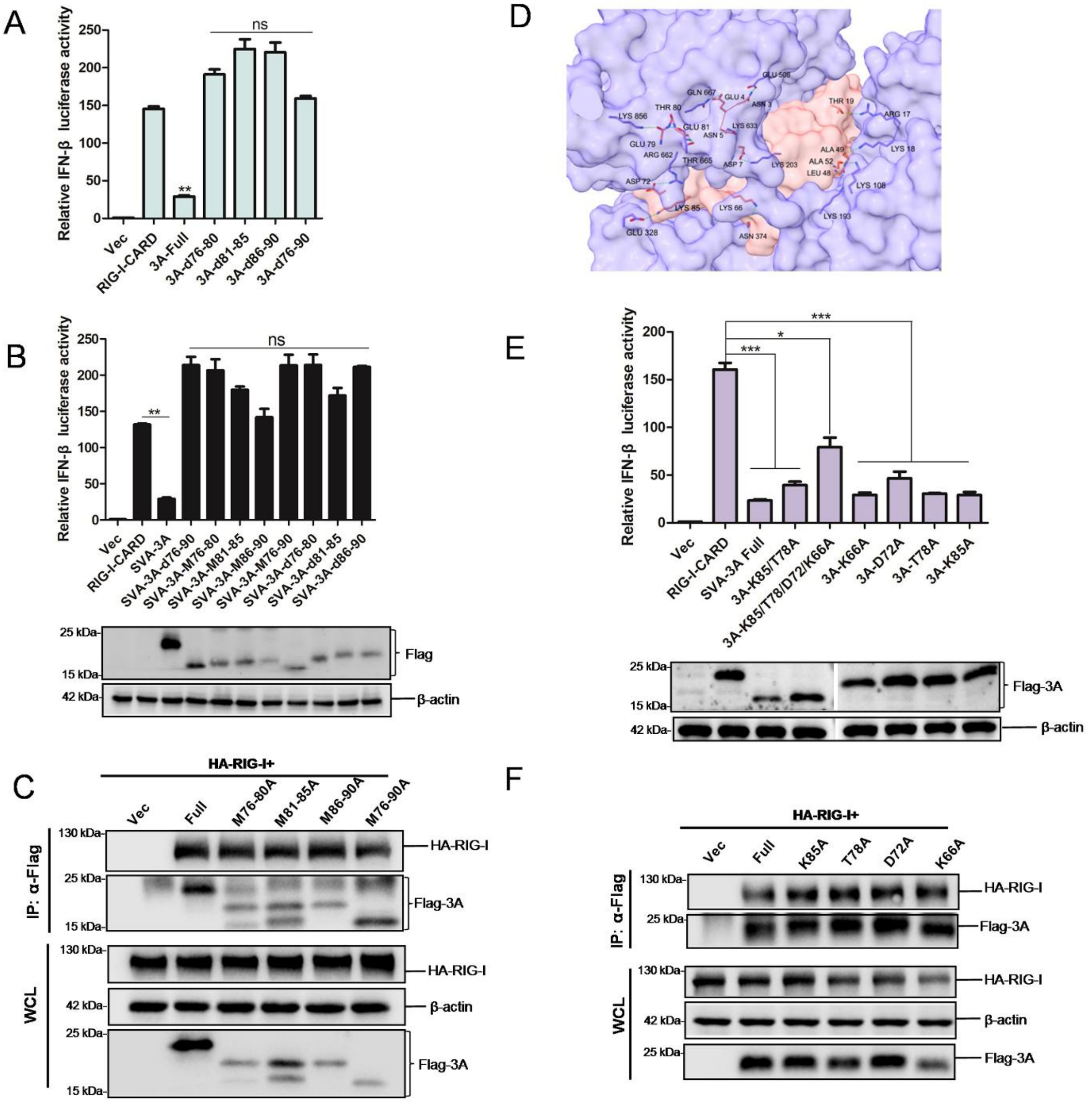
A
Relative IFN-β luciferase activity
ns
**
Vec
RIG-I-CARD
3A-Full
3A-d76-80
3A-d81-85
3A-d86-90
3A-d76-90
B
Relative IFN-β luciferase activity
ns
**
Vec
RIG-I-CARD
SVA-3A
SVA-3A-d76-90
SVA-3A-M76-80
SVA-3A-M81-85
SVA-3A-M86-90
SVA-3A-M76-90
SVA-3A-d76-80
SVA-3A-d81-85
SVA-3A-d86-90
25 kDa-
15 kDa-
Flag
42 kDa-
β-actin
C
HA-RIG-I+
Vec
Full
M76-80A
M81-85A
M86-90A
M76-90A
IP: α-Flag
130 kDa-
HA-RIG-I
25 kDa-
15 kDa-
Flag-3A
WCL
130 kDa-
HA-RIG-I
42 kDa-
β-actin
25 kDa-
15 kDa-
Flag-3A
D
GLU 506
GLN 667
GLU 4
ASN 3
THR 80
THR 19
ARG 17
LYS 856
GLU 81
LYS 633
ASN 5
GLU 79
ARG 662
THR 665
ASP 7
LYS 203
ALA 49
ALA 52
LEU 48
LYS 18
LYS 108
ASP 72
LYS 85
LYS 66
GLU 328
ASN 374
LYS 193
E
Relative IFN-β luciferase activity
***
*
***
Vec
RIG-I-CARD
SVA-3A Full
3A-K85/T78A
3A-K85/T78/D72/K66A
3A-K66A
3A-D72A
3A-T78A
3A-K85A
25 kDa-
15 kDa-
Flag-3A
42 kDa-
β-actin
F
HA-RIG-I+
Vec
Full
K85A
T78A
D72A
K66A
IP: α-Flag
130 kDa-
HA-RIG-I
25 kDa-
Flag-3A
WCL
130 kDa-
HA-RIG-I
42 kDa-
β-actin
25 kDa-
Flag-3A

Supplementary Table 1 The primers of target genes for qPCR used in this study

Target genes (Accession No.)	Primers	Sequences (5′-3′)	Position
h-ISG15	Forward	TGGACAAATGCGACGAACC	224-339
(NM_005101.4)	Reverse	CCCGCTCACTTGCTGCTT	
h-GBP1	Forward	CGAGGGTCTGGGAGATGTAG	294-415
(NM_002053.3)	Reverse	TAGCCTGCTGGTTGATGGTT	
h-ISG54	Forward	AGCAGCCTACGGCAACTAAA	31-206
(NM_001547.5)	Reverse	GCCTCGTTTTGCCCTTTGAG	
h-ISG56	Forward	CCACAAAAAATCACAAGCCATTT	581-675
(NM_001548.5)	Reverse	CAGGGCAAGGAGAACCTTAATATATC	
h-GAPDH	Forward	CGGGAAGCTTGTCATCAATGG	192-549
(NM_002046.7)	Reverse	GGCAGTGATGGCATGGACTG	
h-IFN-β	Forward	GACATCCCTGAGGAGATTAAG	178-260
(NM_002176.4)	Reverse	ATGTTCTGGAGCATCTCATAG	
h-TNF-α	Forward	CCTCTCTGCCATCAAGAGCC	507-688
(NM_000594.4)	Reverse	TCCCAAAGTAGACCTGCCCA	
h-IL-6	Forward	TGACCCAACCACAAATGC	501-605
(NM_000600.5)	Reverse	AGGAACTCCTTAAAGCTGCG	
H-IL-1β	Forward	GCTACGAATCTCCGACCACC	147-347
(NM_000576.3)	Reverse	TCGTGCACATAAGCCTCGTT	
p-IFN-β	Forward	GCTAACAAGTGCATCCTCCAAA	4-80
(NM_001003923.1)	Reverse	AGCACATCATAGCTCATGGAAAGA	
p-TNF-α	Forward	CCACGCTCTTCTGCCTACTGC	134-301
(JF831365.1)	Reverse	GCTGTCCCTCGGCTTTGAC	
p-IL-1β	Forward	GGACATGGAGAAGCGATTCGT	621-738
(NM_001302388.2)	Reverse	GACGGGCTTTTGTTCTGCTT	
p-IL-6	Forward	CCTCTCCGGACAAAACTGAA	143-260
(AF518322.1)	Reverse	TCTGCCAGTACCTCCTTGCT	
p-GAPDH	Forward	TCCCGCCAACATCAAATGGG	237-399
(NM_001206359.1)	Reverse	CACGCCCATCACAAACATGG	
SVA-3D	Forward	AGAATTTGGAAGCCATGCTCT	1202-1279
(KY747510.1)	Reverse	GAGCCAACATAGARACAGATTGC	
